# Efficacy and Genomic Analysis of *HER2*-Mutant Metastatic Triple-Negative Breast Cancer Treated with Neratinib Alone or with Trastuzumab in the SUMMIT Basket Trial

**DOI:** 10.1158/1078-0432.CCR-25-4135

**Published:** 2026-04-06

**Authors:** Komal Jhaveri, Lisa D. Eli, Sara A. Hurvitz, Adam Brufsky, Ron Bose, María de Miguel, Nisha Unni, Sonya Reid, David I. Quinn, Devalingam Mahalingam, Cristina Saura Manich, José Ángel García-Sáenz, Alejandro Martínez-Bueno, Angel Guerrero-Zotano, Olivier Trédan, Hans Wildiers, Georg F. Bischof, Judith Bebchuk, David B. Solit

**Affiliations:** 1 Department of Medicine, Memorial Sloan Kettering Cancer Center, New York, New York.; 2 Weill Cornell Medical College, New York, New York.; 3 Translational Medicine and Diagnostics, Puma Biotechnology, Inc., Los Angeles, California.; 4 Division of Hematology and Oncology, University of Washington School of Medicine and Clinical Research Division, Fred Hutchinson Cancer Center, Seattle, Washington.; 5 University of Pittsburgh Medical Center Magee-Womens Hospital, Pittsburgh, Pennsylvania.; 6 Division of Medical Oncology, Department of Medicine and Siteman Cancer Center, Washington University School of Medicine, St. Louis, Missouri.; 7 START Madrid-CIOCC, HM Sanchinarro Hospital, Madrid, Spain.; 8 Division of Hematology-Oncology, Department of Internal Medicine, Simmons Cancer Center, UT Southwestern Medical Center, Dallas, Texas.; 9 Division of Hematology/Oncology (Breast Oncology), The Vanderbilt-Ingram Cancer Center, Nashville, Tennessee.; 10 Department of Medical Oncology, USC Keck School of Medicine, Norris Comprehensive Cancer Center, Los Angeles, California.; 11 AbbVie Inc., South San Francisco, California.; 12 Robert H. Lurie Comprehensive Cancer Center of Northwestern University, Chicago, Illinois.; 13 Medical Oncology Service, Vall d’Hebron University Hospital, Vall d’Hebron Institute of Oncology, Barcelona, Spain.; 14 Hospital Clínico San Carlos, lnstituto de lnvestigación Sanitaria Hospital Clínico San Carlos (ldlSSC), Madrid, Spain.; 15 Pangaea Biotech, USP, Dexeus University Institute, Barcelona, Spain.; 16 Medical Oncology Department, Instituto Valenciano de Oncologia, GEICAM Breast Cancer Group, Valencia, Spain.; 17 Grupo Español de Investigación en Cáncer de Mama (GEICAM), Madrid, Spain.; 18 Medical Oncology Department, Centre Léon Bérard, Lyon Cedex, France.; 19 Department of Medical Oncology, UZ Gasthuisberg, Leuven, Belgium.; 20 Biostatistics, Puma Biotechnology, Inc., Los Angeles, California.

## Abstract

**Purpose:**

Human epidermal growth factor receptor 2 (*HER2*) mutations occur in 1% to 3% of triple-negative breast cancers (TNBC), representing a novel target for biomarker-directed treatment. In the SUMMIT basket trial (NCT01953926), patients with *HER2*-mutant, metastatic TNBC received neratinib (240 mg/day) or neratinib + trastuzumab (N + T; neratinib 240 mg/day, intravenous trastuzumab 8 mg/kg initially and then 6 mg/kg every 3 weeks). We report final results from the neratinib and N + T TNBC cohorts.

**Patients and Methods:**

Primary endpoint: investigator-assessed objective response rate at first postbaseline tumor assessment (ORR_first_); secondary endpoints included confirmed ORR by investigator, clinical benefit rate (CBR), and progression-free survival (PFS); exploratory endpoint included circulating tumor DNA (ctDNA) collected at baseline, during treatment, and at the end of treatment.

**Results:**

Twenty-seven patients were enrolled between July 2014 and September 2021. Confirmed ORRs were 40% [95% confidence interval (CI), 12.2−73.8] for neratinib (*n* = 10) and 35.3% (95% CI, 14.2−61.7) for N + T (*n* = 17). CBRs were 40% (95% CI, 12.2−73.8) and 47.1% (95% CI, 23−72.2), respectively; median PFS times were 2.89 (95% CI, 0.95−5.52) and 6.24 months (95% CI, 2.10−8.18), respectively. *HER2* mutation variant allele frequencies in ctDNA from patients with response or stable disease decreased upon treatment and increased upon progression. Serial ctDNA sequencing revealed emergence or increase in on-pathway (*ERBB3*) and off-pathway (*KRAS* and *TP53*) mutations. The most common treatment-emergent adverse events were diarrhea, nausea, and constipation.

**Conclusions:**

N + T in patients with *HER2*-mutant metastatic TNBC seemed to prolong responses versus neratinib alone, representing a novel approach for patients with biomarker-defined metastatic TNBC. Based on these and previously published data, neratinib-based combinations are endorsed by the National Comprehensive Cancer Network guidelines for patients with hormone receptor–positive or –negative metastatic breast cancer with activating *HER2* mutations.

*See related commentary by Lloyd et al., p. 3715*

**Translational Relevance**

Treatment options for patients with triple-negative breast cancer (TNBC) were limited until the approval of immunotherapy and antibody–drug conjugates for selected patients. There remains, however, a need for treatments for patients with tumors driven by targetable genomic alterations. In the TNBC cohort of the SUMMIT basket study, neratinib alone or with trastuzumab had encouraging clinical activity in patients with human epidermal growth factor receptor 2 (*HER2*)-mutant TNBC whose disease progressed on other regimens. The addition of trastuzumab to neratinib did not preclude emergence of or increase in on-pathway (*ERBB3*) or off-pathway (*KRAS* and *TP53*) mutations, although no additional *HER2* alterations were detected upon progression in neratinib plus trastuzumab–treated patients in this small dataset. Based on these and earlier data, neratinib-based combinations were added to the National Comprehensive Cancer Network Guidelines for Breast Cancer for patients with hormone receptor–positive or –negative metastatic breast cancer harboring activating *HER2* mutations. Neratinib plus trastuzumab is a novel approach to treating a biomarker-defined population of patients with metastatic TNBC.

## Introduction

Triple-negative breast cancer (TNBC), which represents approximately 15% to 20% of all newly diagnosed breast cancers, is a highly aggressive disease with a poorer prognosis than that of other breast cancer subtypes (1). TNBC is a molecularly heterogeneous disease, defined by a lack of expression of the estrogen and progesterone receptors and a lack of overexpression of human epidermal growth factor receptor 2 (HER2), all of which have enabled the use of targeted therapies in selected patients. Although the advent of immunotherapy and of antibody–drug conjugates has provided novel, targeted treatment options for patients with breast cancer, a biomarker-driven approach to TNBC treatment remains to be developed.

Somatic activating mutations in the *ERBB2* (*HER2*) gene, in the absence of gene amplification or overexpression, are oncogenic drivers in a subset of metastatic breast cancers (MBC; refs. 2–5). *HER2* mutations have been detected in 3%–5% of hormone receptor–positive (HR+) MBC tumors (6, 7) and are further enriched in patients with lobular histology (5%–8%; refs. 8–10). In patients with TNBC, *HER2* mutations have been reported in 1% to 3% of tumors (11, 12) and may represent a novel target for biomarker-directed treatment.

Neratinib is an oral, irreversible, pan-HER tyrosine kinase inhibitor that has demonstrated preclinical and clinical activities against *HER2* mutations (2, 6, 13–18). The original cohorts of the hypothesis-generating SUMMIT basket trial (NCT01953926) evaluated neratinib in patients with *HER2*-mutant metastatic solid tumors, including neratinib as a monotherapy for patients with triple-negative, *HER2*-mutant breast cancer and in combination with fulvestrant for HR+, HER2-negative (HER2–), *HER2*-mutant breast cancer (14). Clinical responses were promising but of short duration, and progression in a subset of patients coincided with the emergence of additional *HER2* mutations and/or amplification of the mutant allele (14). The addition of trastuzumab to the combination of neratinib plus fulvestrant, to achieve dual HER2 blockade with the aim of overcoming the potential resistance arising from increased HER2 signaling, prolonged disease response in patients with HR+, *HER2*-mutant MBC but did not preclude eventual emergence of additional *HER2* alterations coincident with clinical progression (19).

Patients with *HER2*-mutant, metastatic TNBC were enrolled in SUMMIT in two sequential cohorts: patients in the first cohort were treated with neratinib monotherapy, on which partial results were previously reported (14), and those in the second cohort received neratinib + trastuzumab (N + T). Here, we report the final efficacy and safety results for both cohorts. Additionally, utilizing the Memorial Sloan Kettering–Analysis of Circulating Cell-Free DNA to Evaluate Somatic Status (MSK-ACCESS) platform, we assessed serial circulating tumor DNA (ctDNA) genomics to determine whether the potential mechanisms of acquired resistance were similar to or distinct from those previously reported to emerge in patients with HR+, HER2–, *HER2*-mutant MBC who were treated with neratinib + fulvestrant + trastuzumab (N + F + T; ref. 19).

## Patients and Methods

### Study design

The open-label, single-arm, multicohort, multi-tumor, phase II SUMMIT trial was conducted at 57 centers internationally, 17 of which enrolled one or more patients with TNBC. As this was an open-label study, there was no randomization, and no blinding was required. The SUMMIT study has previously been described in detail (13, 14, 19). Patients eligible for inclusion in this cohort were ages ≥18 years, with Eastern Cooperative Oncology Group performance status 0 to 2, histologically confirmed HR–, HER2– (institutionally reported HER2 IHC 0 or 1+, or IHC 2+/FISH test nonamplified) advanced breast cancer with activating *HER2* mutation(s) assessed by local/institutional testing at a commercial or Clinical Laboratory Improvements Act (CLIA)-certified laboratory. Patients had to provide a pretreatment fresh biopsy within 28 days of starting treatment as eligibility required a somatic activating *HER2* mutation, a list of which was derived from characterizations in the scientific literature (PubMed) and/or as identified in two or more cases as detailed in the publicly available Catalogue Of Somatic Mutations In Cancer (RRID: SCR_002260), cBioPortal for Cancer Genomics (RRID: SCR_014555), and OncoKB (RRID: SCR_014782) databases (Supplementary Table S1). Mutations were detected either from formalin-fixed, paraffin-embedded tumor tissue or ctDNA by local testing carried out in a CLIA or equivalent regionally certified laboratory at the time of screening. Central confirmation of *HER2* mutation was carried out retrospectively, and concordance was evaluated. Key exclusion criteria were prior therapy with HER tyrosine kinase inhibitors, cumulative epirubicin dose >900 mg/m^2^ or cumulative doxorubicin dose >450 mg/m^2^, and unstable brain metastases (treated and/or asymptomatic brain metastases were allowed).

The protocol, which complied with the principles of the Declaration of Helsinki, was approved by institutional review boards (IRB) at all participating institutions. All procedures were performed in compliance with relevant laws and institutional guidelines and were approved by the appropriate institutional committee(s). The first IRB approval for the SUMMIT study was received on August 13, 2013; all sites received IRB approval prior to enrolling a patient. Written informed consent was obtained for all patients before carrying out study-related procedures.

### Treatment

The first cohort of patients with TNBC received neratinib monotherapy of 240 mg orally daily. In the second cohort, treatment consisted of neratinib 240 mg orally daily plus trastuzumab 8 mg/kg intravenously followed by 6 mg/kg intravenously every 3 weeks. All patients received mandatory loperamide prophylaxis for the first one to two 28-day cycles and as needed thereafter. Dose escalation was not mandated. Patients were treated until disease progression, unacceptable toxicity, or withdrawal of consent.

### Assessments and endpoints

Tumor response was assessed locally according to Response Evaluation Criteria in Solid Tumors (RECIST) version 1.1 every 8 weeks by computed tomography or magnetic resonance imaging. Patients without RECIST-evaluable disease were evaluated by 18F-fluorodeoxyglucose positron emission tomography (PET; ref. 20). The primary endpoint was objective response rate (ORR) at the first postbaseline tumor assessment (ORR_first_), confirmed or unconfirmed complete response (CR), or partial responses (PR) by RECIST (21) or Positron Emission Tomography Response Criteria in Solid Tumors (PERCIST; ref. 20) per investigator assessment at week 8; CRs or PRs had to be confirmed by repeat tumor assessment using the same method performed ≥4 weeks after the response was initially met. Secondary endpoints were ORR at any assessment, clinical benefit rate [CBR; CR + PR + stable disease (SD) for ≥24 weeks within ± 7-day visit window], duration of response (DOR; defined as the time from when response criteria were first met to progression or death), and progression-free survival (PFS; defined as the interval from day 1 of cycle 1 until the first date on which recurrence, progression, or death due to any cause was documented, censored at the last assessable evaluation or the last evaluation before the initiation of new anticancer therapy if applicable). Exploratory objectives included collection and retrospective evaluation of somatic mutations or gene aberrations using next-generation sequencing (NGS) in the most recent pretreatment tumor biopsy or fresh tumor tissue biopsies at a central laboratory; exploration of genetic modifiers of sensitivity and/or resistance to neratinib using molecular profiling techniques in pretreatment archival and/or fresh tumor specimens and paired normal whole blood; evaluation of cell-free DNA from plasma specimens collected at baseline/screening, during treatment, and upon disease progression to identify *ERBB2* and *EGFR* mutations and other gene aberrations and potential associations with neratinib sensitivity and/or primary/acquired resistance to neratinib or neratinib-containing therapy; and evaluation of potential genes or protein biomarkers conferring neratinib sensitivity and/or primary/acquired resistance to neratinib or neratinib-containing therapy from optional fresh core tumor biopsies during treatment and/or at treatment discontinuation or disease progression.

Central retrospective NGS was performed on pretreatment fresh or archival tumor tissue using MSK-Integrated Mutation Profiling of Actionable Cancer Targets (MSK-IMPACT) and/or on pretreatment ctDNA using MSK-ACCESS. MSK-IMPACT is a hybridization capture-based assay for targeted deep sequencing of all exons and select introns of up to 505 cancer genes using formalin-fixed, paraffin-embedded tumor tissues (22). MSK-ACCESS uses hybridization capture to detect very low frequency somatic alterations in select exons and introns of 129 genes using ctDNA (bioRxiv 2020.06.27.175471; ref. 23).

Adverse events were classified according to Common Terminology Criteria for Adverse Events (version 4.0) from consent until day 28 after discontinuation of study treatment.

### Statistical analysis

Simon's two-stage optimal design was used to determine whether there was sufficient activity to warrant further development of the therapy and to minimize the number of patients exposed to therapy if ineffective. For each treatment arm, using Simon’s two-stage optimal design (with significance level 10% and power of 80%), an ORR (confirmed) of ≤10% per RECIST version 1.1 by local assessment was considered unacceptable (null hypothesis), whereas an ORR (confirmed) of 30% per RECIST v1.1 would merit further study (alternative hypothesis).

In the first stage, seven patients were enrolled in each treatment arm. If one or more responses were observed in the first stage, the second stage would be opened, with 11 additional response-evaluable patients being accrued and randomized for a total of 18 patients in the treatment arm. Enrollment into the TNBC cohort could continue up to 50 patients. The null hypothesis was rejected (for each arm separately) if four or more responses were observed in stage two for each arm.

The ORR_first_, ORR, and CBR were estimated, and their associated two-sided 95% Clopper–Pearson confidence intervals (CI) were determined. The median DOR and median PFS were estimated via Kaplan–Meier methodology with their associated two-sided 95% CIs. The study was not designed to compare efficacy between the neratinib and N + T groups.

All statistical analyses were performed using Statistical Analysis System (version 9.4 and higher; SAS Institute, Inc.; RRID: SCR_008567).

## Results

### Patients

In total, 27 patients with activating *HER2* mutations, 21 assessed via tissue-based and six assessed via liquid-based assays, were enrolled into the SUMMIT TNBC cohorts between July 2014 and September 2021. The first 10 patients received neratinib monotherapy, and 17 subsequent patients received N + T. Patient characteristics are shown in Table 1. See Supplementary Table S2 for a summary of the representativeness of study participants. Most patients were postmenopausal women (*n =* 23; 85.2%) and had visceral disease at enrollment (*n =* 20; 74.1%). Ductal carcinoma was the most common histologic type (*n =* 17; 63%).

**Table 1.** Baseline demographic and clinical characteristics.

Characteristic	Neratinib (*N =* 10)	N + T (*N =* 17)	Total (*N* = 27)
Median age, years (range)	58 (51–70)	62 (39–82)	61 (39–82)
Sex, *n* (%)	​	​	​
Female	9 (90)	16 (94.1)	25 (92.6)
Male	1 (10)	1 (5.9)	2 (7.4)
Race, *n* (%)	​	​	​
White	8 (80)	12 (70.6)	20 (74.1)
Asian	0	0	0
Black or African American	1 (10)	2 (11.8)	3 (11.1)
American Indian or Alaska Native	0	0	0
Other	1 (10)	0	1 (3.7)
Unknown	0	3 (17.6)	3 (11.1)
Menopausal status, *n* (%)	​	​	​
Postmenopausal	9 (90)	14 (82.4)	23 (85.2)
Premenopausal	0	2 (11.8)	2 (7.4)
Not applicable	1 (10)	1 (5.9)	2 (7.4)
ECOG performance status, *n* (%)	​	​	​
0	5 (50)	9 (52.9)	14 (51.9)
1	5 (50)	8 (47.1)	13 (48.1)
Histologic type, *n* (%)	​	​	​
Ductal	8 (80)	9 (52.9)	17 (63)
Lobular	2 (20)	4 (23.5)	6 (22.2)
Other	0	1 (5.9)	1 (3.7)
Unknown	0	3 (17.6)	3 (11.1)
Disease location at enrollment, *n* (%)	​	​	​
Visceral	7 (70)	13 (76.5)	20 (74.1)
Nonvisceral only	3 (30)	4 (23.5)	7 (25.9)
Median time from first metastasis to enrollment, years (range)	1 (0–7)	1.4 (0–8)	1.1 (0–8)
Prior therapies, *n* (%)	​	​	​
Chemotherapy	10 (100)	17 (100)	27 (100)
HER2-directed therapy^a^	1 (10)	2 (11.8)	3 (11.1)
Endocrine therapy^a^	4 (40)	7 (41.2)	11 (40.7)
CDK4/6 inhibitor^a^	3 (30)	3 (17.6)	6 (22.2)
Immunotherapy	0	4 (23.5)	4 (14.8)
Antibody–drug conjugate	0	1 (5.9)	1 (3.7)
Number of prior metastatic regimens, median (range)	2.5 (0–6)	2 (0–7)	2 (0–7)
0, *n* (%)	2 (20)	2 (11.8)	4 (14.8)
1, *n* (%)	0	4 (23.5)	4 (14.8)
2, *n* (%)	3 (30)	3 (17.6)	6 (22.2)
3, *n* (%)	2 (20)	3 (17.6)	5 (18.5)
≥4, *n* (%)	3 (30)	5 (29.4)	8 (29.6)
Number of prior metastatic chemotherapy regimens, median (range)	1 (0–6)	2 (0–7)	1 (0–7)

Abbreviations: CDK4/6, cyclin-dependent kinase 4 and 6; ECOG, Eastern Cooperative Oncology Group.

a All patients who received prior endocrine or HER2-targeted therapy had TNBC confirmed by most recent pathology report prior to enrollment in SUMMIT. All patients who received a CDK4/6 inhibitor also received prior endocrine therapy.

Patients had a median of 2.5 (range, 0–6) prior lines of systemic therapy in the metastatic setting in the neratinib monotherapy cohort and 2.0 (range, 0–7) lines in the N + T cohort (Table 1). Patients in the neratinib monotherapy cohort had a median of 1.0 (range, 0–6) prior lines of chemotherapy in the metastatic setting, and patients in the N + T cohort had a median of 2.0 (range, 0–7) prior lines. All patients had documented HR–, HER2– disease at the time of enrollment into the SUMMIT trial.

Genomic eligibility was based on institutionally reported *HER2* activating mutations, and patient tumor and/or plasma samples were retrospectively centrally confirmed. For cases in which the enrollment and central *HER2* mutations differed (*n =* 2), the centrally detected *HER2* mutation was utilized for the analyses described herein; see Supplementary Table S3 for details about enrollment mutations compared with central retrospective NGS. Twelve unique *HER2* mutations were reported across the 27 patients enrolled in the TNBC cohorts of the SUMMIT trial. The majority had a single missense mutation in the kinase domain (16/27, 59.3%), including V777L (*n =* 5), L755S (*n =* 4), D769Y (*n =* 2), I767M (*n =* 1), R784C (*n =* 1), R849W (*n =* 1), V842I (*n =* 1), and T862A (*n =* 1). Six patients had tumors with an exon 20 insertion mutation (22.2%), specifically G778_P780dup (*n =* 3) and Y772_A775dup (*n =* 3). Three (11.1%) patients harbored a single extracellular domain mutation S310F (*n* = 1) or S310Y (*n* = 2). Two (7.4%) patients harbored dual *HER2* mutations: S310F/V777L (*n =* 1) and V777L/D769Y (*n =* 1). The distribution of *HER2* mutations in these patients with TNBC was similar to that reported previously in the HR+ cohorts of the SUMMIT trial (14, 19) and in publicly available datasets (24, 25). No baseline samples had *HER2* copy-number amplification.

### Efficacy

In total, 90% (9/10) of patients treated with neratinib and 83% (15/17) of those treated with N + T had RECIST version 1.1–evaluable disease at baseline. The three patients without RECIST-evaluable disease were evaluated by 18F-fluorodeoxyglucose PET (20). ORR_first,_ the primary endpoint, was 50% (5/10 patients; 95% CI, 18.7–81.3) in the neratinib group and 29.4% (5/17 patients; 95% CI, 10.3–56) in the N + T group. The ORR at any assessment (secondary endpoint) in the neratinib group was 50% (95% CI, 18.7–81.3) and comprised one CR and four PRs. The ORR in the N + T group was 41.2% (95% CI, 18.4–67.1), including two CRs and five PRs. The confirmed ORR was 40% (95% CI, 12.2–73.8) for patients treated with neratinib and 35.3% (95% CI, 14.2–61.7) for those treated with N + T. The CBRs (secondary endpoint) for neratinib and N + T were 40% (95% CI, 12.2–73.8) and 47.1% (95% CI, 23–72.2), respectively (Table 2).

**Table 2.** Efficacy summary.

Outcome	Neratinib (*N =* 10)	N + T (*N =* 17)
ORR_first_,^a^^,^^b^*n* (%)	​	​
CR at week 8	1 (10)	2 (11.8)
PR at week 8	4 (40)	3 (17.6)
ORR at week 8, % (95% CI)	50 (18.7–81.3)	29.4 (10.3–56)
Overall objective response,^b^*n* (%)	​	​
CR	1 (10)	2 (11.8)
PR	3 (30)	4 (23.5)
ORR, % (95% CI)	40 (12.2–73.8)	35.3 (14.2–61.7)
Best overall response (confirmed or unconfirmed PR or CR), *n* (%)	​	​
CR	1 (10)	2 (11.8)
PR	4 (40)	5 (29.4)
Best overall response (confirmed or unconfirmed PR or CR), % (95% CI)	50 (18.7–81.3)	41.2 (18.4–67.1)
Median DOR,^c^ months (95% CI)	3.78 (3.75–3.88)	6.14 (4.17–9.49)
Clinical benefit,^d^ % *(*95% CI)	40 (12.2–73.8)	47.1 (23–72.2)
Median PFS,^d^ months (95% CI)	2.89 (0.95–5.52)	6.24 (2.10–8.18)

Final data lock date: January 4, 2023. Tumor response based on investigator tumor assessments (RECIST v1.1 or by mPERCIST if RECIST was not available).

Abbreviations: mPERCIST, modified PET RECIST.

a ORR_first_ is the first ORR at the first postbaseline tumor assessment (confirmed or unconfirmed CR or PR by RECIST or PERCIST per investigator assessment at week 8).

b Objective response defined as either a CR or PR as the best overall response that was confirmed no less than 4 weeks after the criteria for response were initially met.

c Kaplan–Meier analysis.

d Clinical benefit defined as confirmed CR, PR, or SD for ≥24 weeks (within ± 7-day visit window).

**Figure 1.**
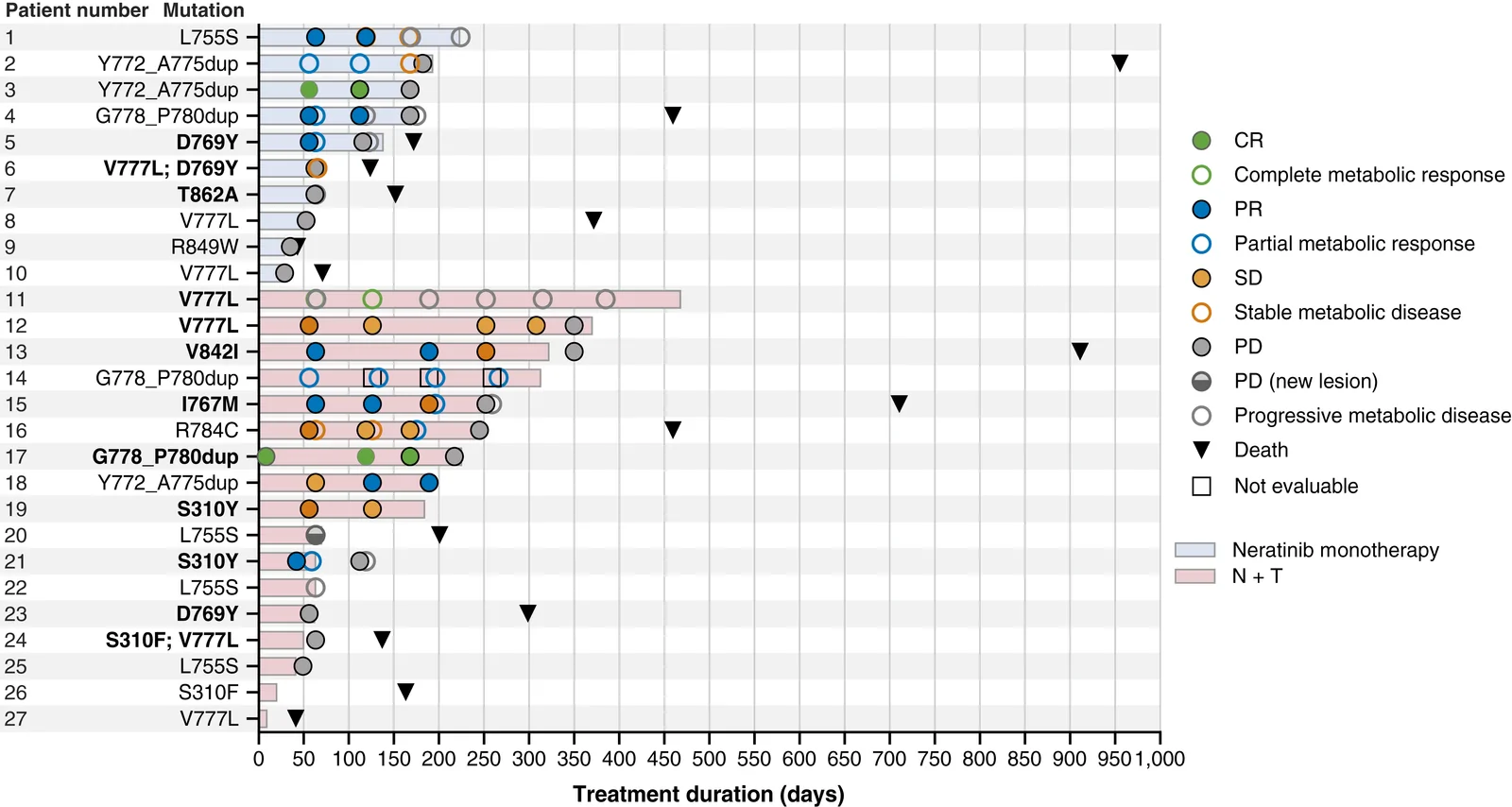
Treatment duration and responses in patients receiving either neratinib monotherapy or N + T. Values in bold indicate patients for whom longitudinal ctDNA sequencing was available. PD, progressive disease.

**Figure 2.**
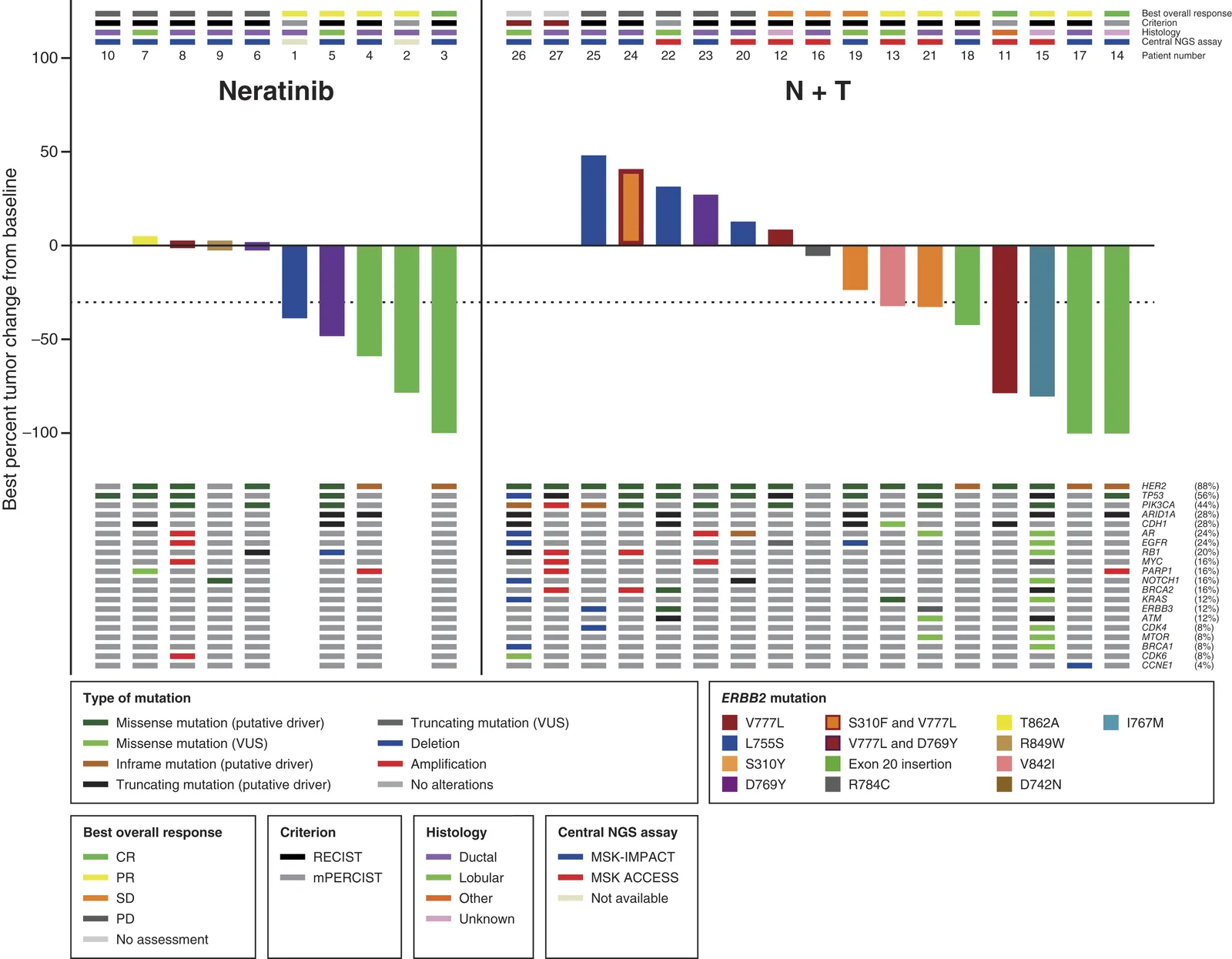
Best change in tumor from baseline and corresponding histology and central biomarker analysis. mPERCIST, modified PET RECIST; PD, progressive disease; VUS, variant of uncertain significance.

Treatment duration and responses are shown in Fig. 1. The median DOR was 3.78 months (95% CI, 3.75–3.88) for patients treated with neratinib and 6.14 months (95% CI, 4.17–9.49) for those treated with N + T. The median PFS was 2.89 months (95% CI, 0.95–5.52) for neratinib and 6.24 months (95% CI, 2.10–8.18) for N + T. Efficacy outcomes are summarized in Table 2 and Figs. 1 and 2.

Efficacy did not seem to depend on histology, as responses were observed in patients with ductal and lobular carcinomas (Fig. 2). In the N + T group, response rates seemed higher in patients who received endocrine therapy over the course of their disease compared with those who did not [*n* = 5/7 (71.4%) and *n* = 1/8 (12.5%), respectively] This observation was less apparent in the neratinib monotherapy group [*n* = 2/4 (50%) and *n* = 2/6 (33.3%), respectively]. Only three patients received prior HER2-directed therapy, exclusively in the (neo)adjuvant setting, with only one evaluable patient in each group: one with progressive disease in the neratinib group and one with a CR in the N + T group. Six of the 12 patients with a clinical response (50%) had *HER2* exon 20 insertion mutation(s), 5 of 12 (41.7%) had a single kinase domain mutation, and 1 of 12 (8.3%) had an S310Y mutation (Fig. 2).

### Central genomic profiling

In total, 25 of 27 patients (92.6%) had tissue and/or plasma available for central retrospective sequencing. Among these 25 patients, 22 (88%) had confirmed *HER2* mutations. None of the three patients whose *HER2* mutation was not detected by central sequencing experienced a response, and none of the three had both tissue and plasma centrally sequenced.

The three most frequently coaltered genes were *TP53* (*n* = 14/25; 56%), *PIK3CA* (*n* = 11/25; 44%), and *ARID1A* (*n* = 7/25; 28%), none of which seemed to preclude response (Fig. 2). Of the 12 patients who experienced a clinical response, 10 had central genomic sequencing: 40% (*n* = 4/10) had a *TP53* comutation, 30% (*n* = 3/10) had a *PIK3CA* comutation, and 40% (*n* = 4/10) had an *ARID1A* comutation. One patient had a tumor with an oncogenic comutation in *ERBB3* (Q809R) and did not experience a response.

### Serial ctDNA sequencing

**Figure 3.**
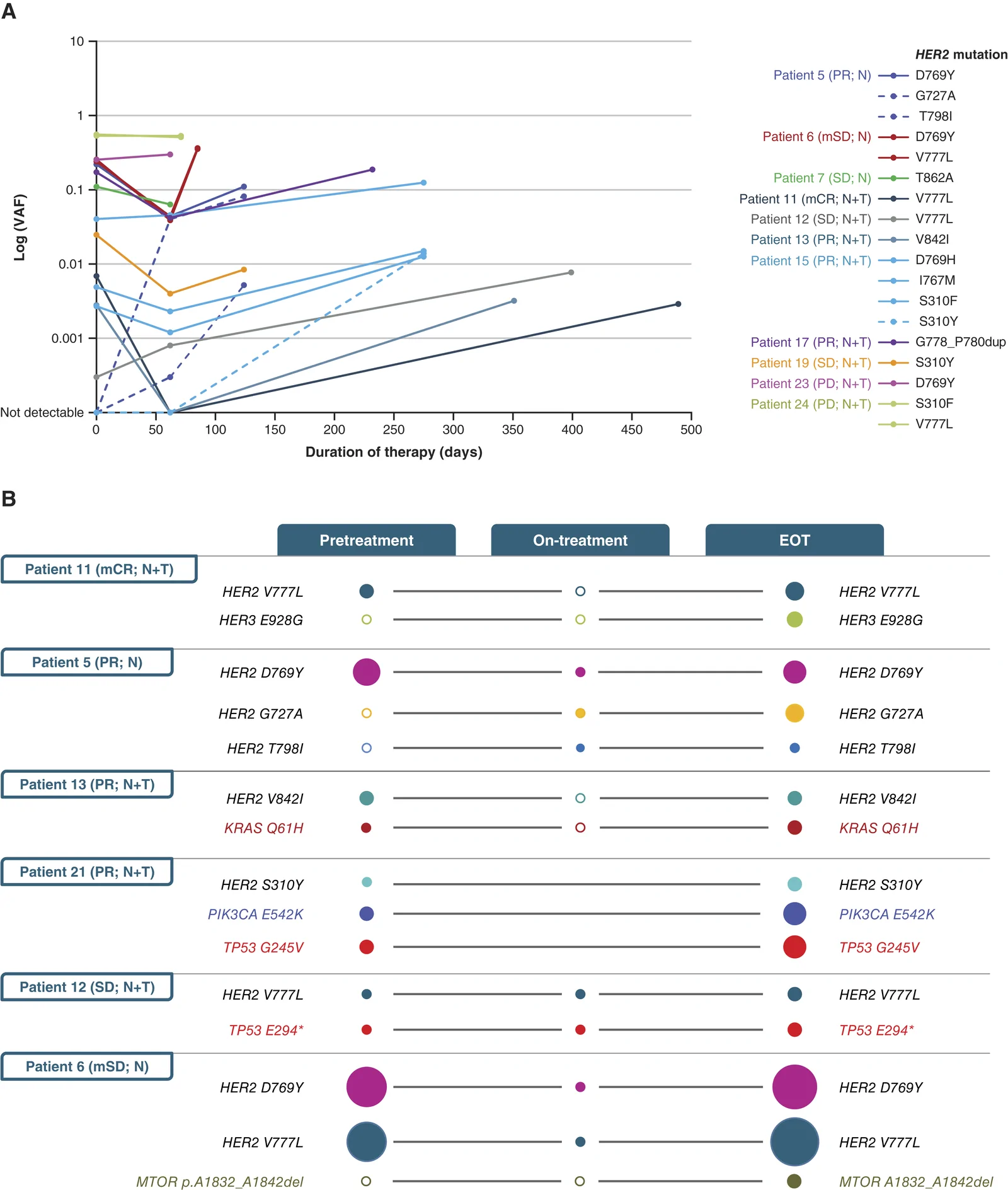
**A,** *HER2* mutation VAF at pretreatment, on-treatment, and EOT stages in patients treated with either neratinib monotherapy or N + T. Note: Patient 21 (PR; treated with N + T) did not have an on-treatment sample and was therefore not included in this figure. **B,** Emergent mutations at progression in ctDNA from patients who experienced a clinical response. Circle sizes are approximately proportional to VAFs. Hollow circles indicate mutation not detectable at that time point. mCR, metabolic CR; mSD, metabolic SD; N, neratinib; PD, progressive disease; T, trastuzumab.

Serial liquid biopsies were assessed by NGS (MSK-ACCESS) to understand genomic changes associated with treatment response and/or resistance. Changes in *HER2* mutation variant allele frequencies (VAF) over the treatment period are shown in Fig. 3A. Five patients with a clinical response had plasma samples with *HER2* mutation detected at baseline, along with on-treatment and end-of-treatment (EOT) samples. *HER2* VAFs decreased upon treatment and reemerged upon progression in all five patients with a clinical response. In one patient treated with neratinib who experienced a PR (patient 5), the *HER2* T798I gatekeeper mutation emerged upon progression, as previously reported (14). An additional *HER2*-sensitizing mutation (S310Y) emerged upon progression in one patient treated with N + T who experienced a PR (patient 15); however, this patient had a very high mutational burden (95 reported mutations), including three intrinsic *HER2* mutations (D769F, I767M, and S310F) and multiple other apparently emergent mutations, and thus the relevance of the *HER2* S310Y mutation is unclear.

Three patients with SD or metabolic SD had plasma samples with *HER2* mutation detected at baseline, along with successfully sequenced on-treatment and EOT samples (patients 6, 12, and 19). *HER2* VAFs decreased upon treatment in two of the three patients with SD or metabolic SD, and reemergence was observed in those patients upon progression. Three patients with progressive disease had plasma samples with *HER2* mutation detected at baseline and an EOT sample (patients 7, 23, and 24). *HER2* VAFs decreased minimally (patients 7 and 24) or increased minimally (patient 23) for these patients.

Emergent mutations detected at progression were seen in two patients treated with neratinib and four patients treated with N + T who experienced an initial response or SD (Fig. 3B). Emergent mutations were defined as either (i) not detected at baseline and detected at EOT or (ii) detected at baseline and increased on treatment in parallel with a decrease in *HER2* mutation VAF or less of an increase, proportionally, compared with the change in *HER2* mutation VAF. On-pathway emergent mutations included the *HER2* gatekeeper T798I, the oncogenic mutations *HER3* E928G and *PIK3CA* E542K, and a short in-frame *MTOR* deletion. The patient with the emergent *HER3* E928G mutation (patient 11) was the only one among the five patients with a clinical response who had received prior anti-HER2 therapy (trastuzumab and pertuzumab). Off-pathway mutations included *KRAS* Q61H, *TP53* E294*, and *TP53* G245V. The VAFs for both *TP53* mutations increased over the treatment course, whereas the *KRAS* Q61H mutation initially disappeared and then reemerged with a higher VAF than observed at baseline.

### Safety

The most common treatment-emergent adverse events were diarrhea, nausea, and constipation, as shown in Table 3. Diarrhea of any grade was reported in 80% (*n* = 8/10) of patients treated with neratinib and in 100% (*n* = 17/17) of patients treated with N + T. Grade 3 diarrhea occurred in 20% (*n* = 2/10) of patients who received neratinib and 17.6% (*n* = 3/17) of those who received N + T. In all patients, grade 3 diarrhea occurred early after treatment initiation (median time to grade 3 diarrhea, 7 days; IQR, 6–9 days) and was of short duration (cumulative median, 2.0 days; IQR, 1–5 days). Among the 25 patients who experienced diarrhea, four were managed with temporary dose interruption and three with dose reduction; none of these patients discontinued treatment. One patient who was treated with N + T discontinued on day 64 as the result of a seizure that was attributed to brain metastases and not attributable to neratinib or trastuzumab.

**Table 3.** Most common treatment-emergent adverse events^a^.

Event, *n* (%)	Neratinib (*N =* 10)		N + T (*N =* 17)	
	All grades	Grade 3/4	All grades	Grade 3/4
Diarrhea	8 (80)	2 (20)	17 (100)	3 (17.6)
Nausea	7 (70)	0	8 (47.1)	1 (5.9)
Constipation	6 (60)	0	6 (35.3)	0
Fatigue	4 (40)	0	4 (23.5)	2 (11.8)
Vomiting	3 (30)	0	9 (52.9)	1 (5.9)
Decreased appetite	3 (30)	0	4 (23.5)	0
Abdominal pain	3 (30)	0	2 (11.8)	1 (5.9)
Pyrexia	2 (20)	0	1 (5.9)	0
Anemia	1 (10)	1 (10)	4 (23.5)	0
Hypokalemia	0	0	4 (23.5)	1 (5.9)

a Includes any treatment-emergent adverse event occurring in ≥20% of patients in either cohort in order of prevalence in the neratinib cohort.

## Discussion

Treatment options for patients with TNBC have been limited until recently, when the development and introduction of immunotherapy and antibody–drug conjugates improved outcomes for selected patients (26). There remains, however, a need for effective, targeted treatments for the management of patients whose tumors may be driven by targetable genomic alterations. In the TNBC cohort of the SUMMIT basket study, reported herein, treatment with neratinib either alone or in combination with trastuzumab had encouraging clinical activity in patients with *HER2*-mutant TNBC whose disease had progressed on other regimens, suggesting a potential role for neratinib in this setting.

In the TNBC cohort, patients treated with neratinib had a confirmed ORR of 40%, median DOR of 3.78 months, CBR of 40%, and median PFS of 2.89 months; those treated with N + T had a confirmed ORR of 35.3%, median DOR of 6.14 months, CBR of 47.1%, and median PFS of 6.24 months. Responses to neratinib or N + T were histology-independent, occurring in patients with ductal and lobular histologies. Triple-negative intralobular carcinoma (TN-ILC) is rare, reported in <2% of ILC cases (27), with high overlap of the luminal androgen receptor subtype and TN-ILC. Of note, *HER2* mutations have been reported in up to 20% of TN-ILC (11, 27). In the present study, six of the 27 enrolled patients (22%) had TN-ILC harboring *HER2* mutations, consistent with the enrichment of lobular carcinoma in *HER2*-mutant TNBC. Although caveated due to the lack of cohort randomization, patients treated with N + T seemed to experience longer time to progression than those who received neratinib monotherapy. This encouraging clinical activity supports the previously reported benefit of N + F + T in SUMMIT patients with HR+, *HER2*-mutant MBC (19), data that are consistent with the hypothesis that dual HER2 targeting may address the previously reported emergence of additional *HER2* mutations and amplifications as the dominant mechanism of acquired resistance to neratinib-containing regimens in the SUMMIT HR+ breast cancer cohorts and MutHER patient tumors (18, 19). These data are in line with the activity observed for patients with *HER2*-mutant MBC in the basket study of tucatinib plus trastuzumab in HER2-altered solid tumors (NCT04579380), in which an ORR of 42% was reported in 31 patients (28).

Although based on small numbers, patients whose tumors harbored *HER2* exon 20 insertions seemed to have the greatest responses to neratinib or N + T. Responses were also observed in patients with kinase domain missense mutations. No pattern of association was observed between response to neratinib or N + T and any comutation, including but not limited to *PIK3CA* or *TP53*. Serial ctDNA sequencing findings, although limited by the small number of patients with sufficient samples, suggest that the addition of trastuzumab to neratinib in patients with *HER2*-mutant metastatic TNBC, despite deepening and prolonging responses, did not preclude eventual emergence of either on-pathway (*ERBB3*) or off-pathway (*KRAS* and *TP53*) mutations, with the *KRAS* mutation initially disappearing and ultimately reemerging with an increased VAF. Similar observations regarding emergent on- or off-pathway mutations have been made for the N + F + T–treated HR+, *HER2*-mutant cohort of the SUMMIT trial (19). Future investigations may evaluate the clinical utility of sequencing therapies targeted at mutations acquired in response to neratinib-based therapy in patients with *HER2*-mutant metastatic TNBC.

Recent studies have examined the role of HER2-directed therapy in patients without HER2 amplification, overexpression, or both, i.e., the HER2-low cancers, which may be targetable but were previously considered difficult to treat with conventional chemotherapy. Treatment of patients with HER2-low MBC with trastuzumab deruxtecan (T-DXd) versus physician’s choice in the phase III DESTINY-Breast04 trial revealed increased PFS and overall survival in the T-DXd arm (29). In the subgroup of 58 efficacy-evaluable patients with HR–, HER2-low disease in that trial, those treated with T-DXd experienced a PFS of 8.5 months, compared with 2.9 months for those who received physician’s choice of treatment (29). Interestingly, the combination of N + T demonstrated preclinical efficacy in a panel of HER2-low cell lines and in patient-derived organoids (30). Furthermore, the combination of neratinib and ado-trastuzumab emtansine was synergistic in preclinical models (bioRxiv 2023.12.19.572069), likely due to neratinib-induced internalization of HER2 and the bound drug/payload (bioRxiv 2023.12.19.572069; ref. 31).

Following on from these studies, which suggest that the combination of neratinib plus ado-trastuzumab emtansine warrants investigation, a safety and dose-finding study evaluating the combination of neratinib and T-DXd in patients with HER2-altered solid tumors is underway (NCT05372614). Whether these agents should be utilized in combination or may be better if used in sequence to further improve outcomes for *HER2*-mutant, HER2-low HR– MBC remains to be determined.

Some limitations of this study warrant consideration. The sample size was small, and there was a lack of randomization, with no direct comparisons between neratinib monotherapy and N + T. Furthermore, not all patients had plasma available for serial ctDNA analysis, so additional mechanisms of acquired resistance/emergent mutations may not be represented. Additionally, genomic eligibility for the study was not centrally assessed, and both tissue- and liquid-based NGS approaches were used. Although the general concordance between the two technologies is considerable, methodologic differences exist that could explain why *HER2* mutations in three patients were not detected by central sequencing. Future studies should adopt a single-technology, centralized approach to inform *HER2* mutational status.

In conclusion, the addition of trastuzumab to neratinib in patients with *HER2*-mutant metastatic TNBC did not preclude eventual emergence or increase of either on-pathway (*ERBB3*) or off-pathway (*KRAS* and *TP53*) mutations, although responses seemed to be deepened and prolonged with the combination. In contrast to observations in N + F + T–treated patients with HR+, *HER2*-mutant disease, no additional *HER2* alterations were detected upon progression in N + T–treated patients with metastatic TNBC in this small dataset. Based on these data and previously published results (18, 19), neratinib-based combinations have been included in the National Comprehensive Cancer Network Guidelines in Oncology for Breast Cancer for patients with HR+ or HR− MBC harboring an activating *HER2* mutation (32).

## Supplementary Material

Supplementary Data1 Supplementary Tables 1-3.

## Data Availability

The authors declare that the data supporting the findings of this study are available within the article. All available centralized patient-level genomic data are available on cBioPortal.org (https://www.cbioportal.org/study/summary?id=summit_tnbc_2026). Raw sequencing data are not publicly available to comply with patient consent forms and IRB/Health Insurance Portability and Accountability Act requirements but can be made available to qualified researchers upon reasonable request to Komal Jhaveri (jhaverik@mskcc.org), contingent upon institutional approval and the execution of a Data Use Agreement. Qualified researchers and study participants may submit requests for other study documentation and clinical trial data to clinicaltrials@pumabiotechnology.com for consideration.
