# Supplementary material for: Efficacy and Genomic Analysis of HER2-Mutant Metastatic Triple-Negative Breast Cancer Treated with Neratinib Alone or with Trastuzumab in the SUMMIT Basket Trial
Source: Clin Cancer Res. 2026 Apr 6;32(17):3735–45. doi: 10.1158/1078-0432.CCR-25-4135 (PMC13530984; doi:10.1158/1078-0432.CCR-25-4135)
Supplement: Supplementary file 1 [file ccr-25-4135_supplementary_data1_suppds1.docx]

# Efficacy and Genomic Analysis of HER2-Mutant Metastatic Triple-Negative Breast Cancer Treated with Neratinib Alone or with Trastuzumab in the SUMMIT Basket Trial

**Authors:** Komal Jhaveri^1^, Lisa D. Eli^2^, Sara A. Hurvitz^3^, Adam Brufsky^4^, Ron Bose^5^, Maria de Miguel^6^, Nisha Unni^7^, Sonya Reid^8^, David I. Quinn^9,10^, Devalingam Mahalingam^11^, Cristina Saura^12^, José Ángel García-Sáenz^13^, Alejandro Martínez-Bueno^14^, Angel Guerrero-Zotano^15,16^, Olivier Trédan^17^, Hans Wildiers^18^, Georg F. Bischof^19^, Judith Bebchuk^19^, David B. Solit^1^

# Extended Data

**Supplementary Table S1. Eligible *ERBB2* activating mutations**

| **One-letter amino acid nomenclature** | **Three-letter amino acid nomenclature** |
| --- | --- |
| G309A | Gly309Ala |
| G309E | Gly309Glu |
| S310F | Ser310Phe |
| S310Y | Ser310Tyr |
| S653C | Ser635Cys |
| V659E | Val659Glu |
| G660D | Gly660Asp |
| V697L | Val697Leu |
| T733I | Thr733Ile |
| L755S | Leu755Ser |
| L755P | Leu755Pro |
| L755_T759del | Leu755_Thr759del |
| I767M | Ile767Met |
| D769Y | Asp769Tyr |
| D769H | Asp769His |
| D769N | Asp769Asn |
| Y772_A775dup | Tyr772_Ala755dup |
| G776C | Gly776Cys |
| G776V | Gly776Val |
| G776VinsC/VGC/other | Gly776insCys/other |
| Other exon 20 insertions | N/A |
| V777L | Val777Leu |
| G778_P780dup | Gly778_Pro780dup |
| L841V | Leu841Val |
| V842I | Val842Ile |
| L869R | Leu869Arg |
| L866M | Leu869Met |
| H878Y | His878Tyr |
| R896C | Arg896Cys |
| T862A | Thr862Ala |
| R849W | Arg849Trp |
| R784C | Arg784Cys |

Eligible *ERBB2* mutations were derived from characterizations in the scientific literature (PubMed) and/or as identified in two or more cases as detailed in publicly available databases (COSMIC, cBioPortal for Cancer Genomics, OncoKB^TM^).

**Supplementary Table S2. Representativeness of Study Participants**

| **Cancer type** | **Triple-negative breast cancer** |
| --- | --- |
| Sex | TNBC, like all other subtypes of breast cancer, is a predominantly female disease and is rare in men. Male breast cancer represents <1% of all breast cancers diagnosed each year (Eric S, Niba JO. Cureus. 2025;17:e87611). Ghani and colleagues identified 66 men with TNBC in their analysis of SEER 18 data (Ghani S, et al. Breast J. 2020;26;1748–55) compared with 238,848 women with TNBC (Zhang W, et al. Front Public Health. 2022;10:1058722). |
| Age | Scott and colleagues conducted a population-based study from the United States Cancer Statistics database between 2010 and 2014. The mean age of all patients was 61.8 years. Comparing age groups, with the reference being women aged 50–64 years, women aged <40 years had the highest odds of TNBC diagnosis (OR, 1.95; 95% CI, 1.90−2.01), whereas those aged ≥75 years had the lowest odds (OR, 0.75; 95%CI, 0.73−0.76) (Scott LC, et al. Cancer. 2019;125:3412−17). |
| Race/ethnicity | Scott and colleagues also reported on racial disparities in their analysis of the United States Cancer Statistics database between 2010 and 2014. In unadjusted analyses, non-Hispanic Black women, who accounted for 21% of the TNBC patient population (OR, 2.27; 95% CI, 2.23−2.31), and Hispanic women, who accounted for 9% of the population (OR, 1.22; 95% CI, 1.19−1.25), had higher odds of diagnosis than non-Hispanic white women, who accounted for 66% of the population. |
| Geography | TNBC accounts for about 10−15% of all breast cancers. In the USA, an estimated 238,848 patients were diagnosed with TNBC between 2011 and 2019 (Zhang W, et al. Front Public Health. 2022;10:1058722). |
| Other considerations | Underrepresentation in trials limits evaluation of the impact of racial/ethnic- or ancestry-based differences in efficacy and toxicity.  Biomarker studies are typically undertaken as part of larger clinical trials, which are not generally representative of the general breast cancer population, as demonstrated above. |
| Overall representativeness of this study | The age distribution of our study is similar to the average age distribution of TNBC (median age 62; range 39−82 years).  This study included more non-Hispanic white patients than are seen in the general population of people with TNBC in the USA. Men accounted for <10% of the patient population in the present study, higher than reported in previous analyses of cancer registries. |

Abbreviations: CI, confidence interval; OR, odds ratio; SEER, National Cancer Institute Surveillance, Epidemiology, and End Results Program; TNBC, triple-negative breast cancer.

Supplementary Table S3. Enrollment mutations and those identified in central retrospective next-generation sequencing of ctDNA

| Patient | Enrollment biopsy type | Enrollment HER2 mutation | Central NGS tissue HER2 mutation | Central NGS baseline plasma mutation |
| --- | --- | --- | --- | --- |
| Patient 1 | Tissue | L755S | NA | NA |
| Patient 2 | Tissue | Y772_A775dup | NA | NA |
| Patient 3 | Tissue | Y772_A775dup | Y772_A775dup | NA |
| Patient 4 | Tissue | G778_P780dup | G778_P780dup | NA |
| Patient 5 | Tissue | D769Y | D769Y | NA |
| Patient 6 | Tissue | V777L; D769Y | V777L, D769Y | NA |
| Patient 7 | Tissue | T862A | NA | T862A |
| Patient 8 | Tissue | V777L | V777L | NA |
| Patient 9 | Plasma | R849W | ND | NA |
| Patient 10 | Tissue | V777L | NA | ND |
| Patient 11 | Plasma | V777L | NA | V777L |
| Patient 12 | Plasma | V777L | NA | V777L |
| Patient 13 | Tissue | V842I | NA | V842I |
| Patient 14 | Tissue | G778_P780dup | G778_P780dup | NA |
| Patient 15 | Tissue | I767M | NA | S310F, S310Y, I767M, D769H |
| Patient 16 | Tissue | R784C | NA | ND |
| Patient 17 | Plasma | G778_P780dup | G778_P780dup | G778_P780dup |
| Patient 18 | Plasma | Y772_A775dup | Y772_A775dup | ND |
| Patient 19 | Tissue | S310Y | S310Y | S310Y |
| Patient 20 | Tissue | L755S | NA | ND |
| Patient 21 | Plasma | D742N | NA | S310Y |
| Patient 22 | Tissue | L755S; V842I | NA | L755S |
| Patient 23 | Tissue | D769Y | D769Y | D769Y |
| Patient 24 | Tissue | S310F; V777L | S310F, V777L | S310F, V777L |
| Patient 25 | Tissue | L755S | L755S | ND |
| Patient 26 | Tissue | L755S | S310F | ND |
| Patient 27 | Tissue | V777L | V777L | V777L |

Abbreviations: ctDNA, circulating tumor DNA; HER2, human epidermal growth factor receptor 2; NA, sample not available; ND, central NGS did not detect an *HER2* mutation in the sample; NGS, next-generation sequencing.
